# IGF2BP3 promotes adult myocardial regeneration by stabilizing *MMP3* mRNA through interaction with m6A modification

**DOI:** 10.1038/s41420-023-01457-3

**Published:** 2023-05-15

**Authors:** Simeng Li, Siman Shen, Hao Xu, Shuyun Cai, Xiaodong Yuan, Changsen Wang, Xiaojun Zhang, Suyun Chen, Jianning Chen, De-Li Shi, Liangqing Zhang

**Affiliations:** 1 Department of Anesthesiology, Affiliated Hospital of Guangdong Medical University, Zhanjiang, China; 2 Zhanjiang Key Laboratory of Organ Function Injury and Protection, Affiliated Hospital of Guangdong Medical University, Zhanjiang, China; 3 Guangdong Provincial Key Laboratory of Autophagy and Major Chronic Non-communicable Diseases, Affiliated Hospital of Guangdong Medical University, Zhanjiang, China; 4 Laboratory of Developmental Biology, CNRS-UMR7622, Institut de Biologie Paris-Seine (IBPS), Sorbonne University, 75005 Paris, France

**Keywords:** Cardiovascular diseases, Epigenetics

## Abstract

Myocardial infarction that causes damage to heart muscle can lead to heart failure. The identification of molecular mechanisms promoting myocardial regeneration represents a promising strategy to improve cardiac function. Here we show that IGF2BP3 plays an important role in regulating adult cardiomyocyte proliferation and regeneration in a mouse model of myocardial infarction. IGF2BP3 expression progressively decreases during postnatal development and becomes undetectable in the adult heart. However, it becomes upregulated after cardiac injury. Both gain- and loss-of-function analyses indicate that IGF2BP3 regulates cardiomyocyte proliferation in vitro and in vivo. In particular, IGF2BP3 promotes cardiac regeneration and improves cardiac function after myocardial infarction. Mechanistically, we demonstrate that IGF2BP3 binds to and stabilizes *MMP3* mRNA through interaction with N^6^-methyladenosine modification. The expression of MMP3 protein is also progressively downregulated during postnatal development. Functional analyses indicate that MMP3 acts downstream of IGF2BP3 to regulate cardiomyocyte proliferation. These results suggest that IGF2BP3-mediated post-transcriptional regulation of extracellular matrix and tissue remodeling contributes to cardiomyocyte regeneration. They should help to define therapeutic strategy for ameliorating myocardial infarction by inducing cell proliferation and heart repair.

## Introduction

Percutaneous coronary interventions have been widely used for treatment of acute myocardial infarction (MI) complicated by cardiogenic shock. Nevertheless, the prognosis remains poor in patients suffering from this condition [1]. Induction of endogenous myocardial regeneration represents a promising strategy for management of cardiogenic shock. It has been reported that certain vertebrate species, such as zebrafish, can fully regenerate the injured myocardium by activating several signaling pathways, such as Notch and BMP, to induce the proliferation of spared cardiomyocytes (CMs) in a non-cell autonomous manner [2, 3**]**. In neonatal mice, the heart can also undergo regeneration after partial surgical resection, but this ability is rapidly lost a few days after birth [4]. Severe MI caused by coronary occlusion in newborn humans can be recovered to long-term normal cardiac function within a few weeks, suggesting that infants may possess the intrinsic ability of myocardial regeneration [5].

Mammalian CMs irreversibly exit cell cycle shortly after birth, which prevents cardiac regeneration. However, after MI, adult hearts can form new CMs through dedifferentiation, proliferation, and redifferentiation [6]. Previous studies have shown that numerous signal transduction pathways and transcription factors regulate the proliferation of adult mammalian CMs [7–11]. Several proteins are involved in the control of cell cycle entry in the postnatal heart. Constitutive expression of Cyclin A2 in the myocardium can improve restricted ventricular dilation and sustain cardiac function in mice after infarction by inducing the formation of new CMs in the infarct area. In this situation, there is resumption of proliferation in the peri-infarct myocardium, promoted by an increased DNA synthesis and mitotic index, which allows mice to trigger regenerative response after heart infarction [12]. Overexpression of Cyclin A2 in infarcted pig hearts can also initiate a regenerative response [13], while transgenic expression of Cyclin D2 in mice initiates DNA synthesis and leads to infarct regression in CMs [14]. Upregulation of cyclin-dependent kinase 1 (CDK1) can effectively induce division of post-mitotic CMs in mice, rats and humans, which significantly improves cardiac function following acute or subacute MI [15]. Thus, strategies that induce cell cycle re-entry and proliferation of CMs could promote cardiac regeneration in mammals [16**–**18].

N6-methyladenosine (m6A) is the most abundant modification present in eukaryotic mRNAs, which controls gene expression by affecting multiple steps of mRNA metabolism including alternative splicing, subcellular localization, translation, and stability [19]. Emerging evidence indicates that m6A modification is a dynamic and reversible process involving methyltransferases (METTL3, METTL14, and WTAP), demethylases (FTO and ALKBH5), and RNA-binding proteins including insulin-like growth factor 2 mRNA-binding proteins (IGF2BPs), YTHDF1–3, and YTHDC1–2 [20]. IGF2BPs function as m6A readers and target thousands of mRNA transcripts through recognition of the GG(m6A)C consensus sequence [21]. Therefore, IGF2BPs exert their biological functions through post-transcriptional regulation of gene expression, promoting the stability and storage of its target mRNAs under normal and stressed conditions [22]. Interestingly, the expression of IGF2BP3 shows sharp decline from postnatal day 1 (P1) to postnatal day 8 (P8), and is absent in the adult heart. Overexpression of IGF2BP3 can promote the proliferation of CMs in the neonatal mice [23]. However, molecular mechanisms by which IGF2BP3 regulates CM proliferation and cardiac regeneration are incompletely understood. In particular, whether IGF2BP3 can promote adult myocardial regeneration and improve cardiac function after MI remains unknown.

In this study, we report that IGF2BP3 functions upstream of matrix metalloproteinase 3 (MMP3) to enhance mitosis and trigger proliferation of adult CMs, thus promoting cardiac regeneration and improving cardiac function after cardiac injury. Mechanistically, IGF2BP3 stabilizes *MMP3* mRNA through interaction with m6A modification to increase MMP3 protein level. We further demonstrate that both IGF2BP3 and MMP3 promote cell cycle progression to induce CM proliferation. Therefore, our findings reveal a critical role for IGF2BP3 and MMP3 in adult cardiac regeneration.

## Results

### Upregulation of IGF2BP3 expression after myocardial infarction

**Fig. 1:**
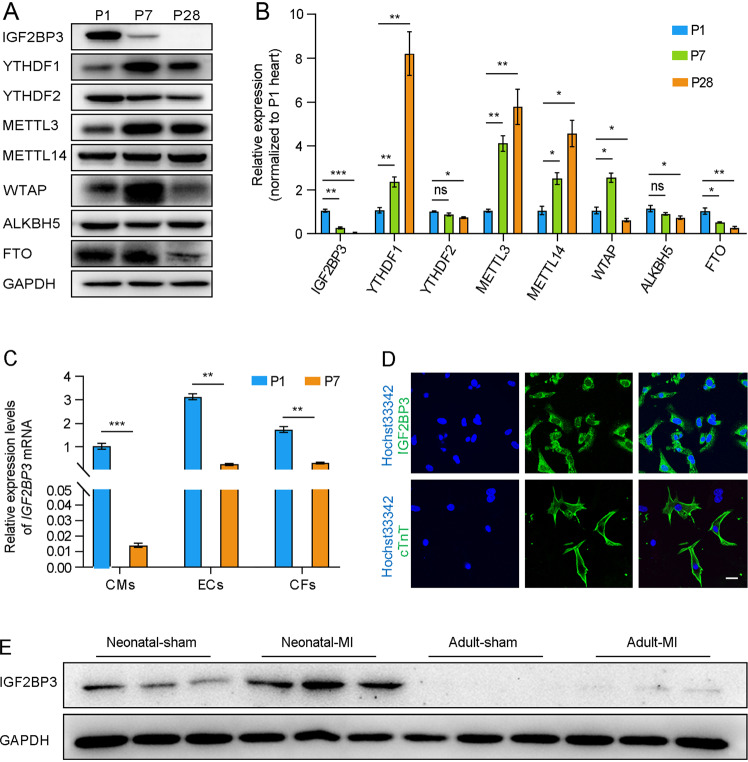
IGF2BP3 expression during mouse postnatal cardiac development and after MI. **A** Western blot analysis of proteins related to m6A modification. Note the strong decrease and absence of IGF2BP3 expression at P7 and P28, respectively. **B** RT-qPCR analysis compares the relative expression of genes associated with m6A modification. Values in P1 conditions are set to 1 as a reference, after normalization to GAPDH. Data are the mean ± s.e.m. from three independent samples (*P < 0.05; **P < 0.01; ***P < 0.001; ns, not significant). **C** RT-qPCR analysis compares *IGF2BP3* expression levels in CMs, ECs, and CFs from P1 and P7 hearts. The expression levels of *IGF2BP3* in P1 conditions are set to 1 as a reference, after normalization to GAPDH. Data are the mean±s.e.m. from three independent samples (**P < 0.01; ***P < 0.001). **D** Immunostaining shows cytoplasmic localization of IGF2BP3 in cultured CMs, which were confirmed by the expression of cardiac Troponin T (cTnT). Scale bar: 20 µm. **E** Western blot analysis of cardiac IGF2BP3 expression at day 7 post-MI in neonatal and adult mice.

By methylated RNA immunoprecipitation (MeRIP)-sequencing (MeRIP-seq) and RNA-sequencing (RNA-seq), we previously identified differential expression of m6A-related methyltransferases, demethylases and reader proteins in the heart between P1, P7 and P28 mice [24]. In the present study, we sought to validate these data by western blot analysis. Although there was a complex pattern in the expression of m6A-related proteins, we found that the expression of IGF2BP3 in the heart progressively decreased during postnatal development and became undetectable in the P28 heart. By contrast, the cardiac expression of YTHDF1, METTL3 and METTL14 was upregulated in P7 and P28 mice compared with P1 mice (Fig. 1A). This result was further confirmed by RT-qPCR analysis (Fig. 1B), suggesting a postnatal downregulation in the transcription of *IGF2BP3* gene. Moreover, RT-qPCR analysis revealed that this temporal downregulation of IGF2BP3 was most prominent in CMs, although the expression level was also significantly decreased in endothelial cells (ECs) and cardiac fibroblasts (CFs) isolated at P7 (Fig. 1C). In isolated P1 CMs, immunofluorescence staining showed a predominant cytoplasmic localization of IGF2BP3 protein (Fig. 1D), suggesting that it mostly functions in the cytoplasm. Interestingly, IGF2BP3 expression was strongly induced after MI in neonatal mice, but it was also weakly detectable following MI in adult mice (Fig. 1E). In the latter situation, IGF2BP3 protein was mainly detected in the border zone of the MI heart (Supplementary Fig. S1A), and showed highest expression level at 1 day post-infarction (Supplementary Fig. S1B). These observations suggest that *IGF2BP3* is a cardiac injury-responsive gene and may play a role in adult cardiac regeneration.

### IGF2BP3 regulates cardiomyocyte proliferation in vitro

**Fig. 2:**
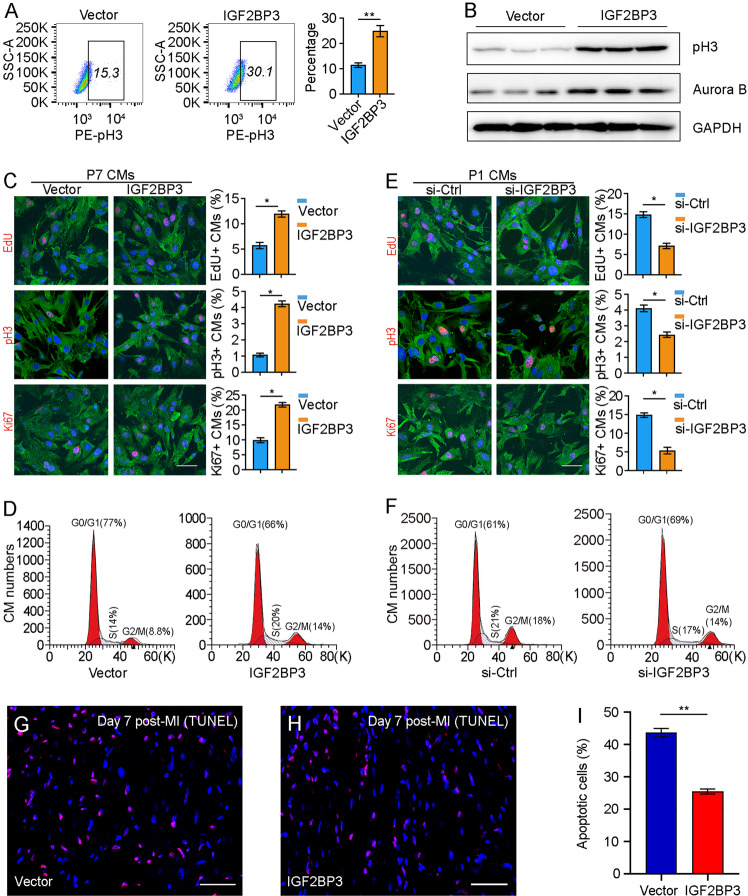
IGF2BP3 promotes proliferation of postnatal CMs in vitro. **A** Flow cytometry analysis shows the increase of pH3-positive P7 CMs overexpressing IGF2BP3. **B** Western blot analysis of pH3 and Aurora B proteins in P7 CMs overexpressing IGF2BP3. **C** Effects of IGF2BP3 overexpression on CM proliferation. Representative images and statistical analyses of P7 CMs stained by cell proliferation markers. For each condition, 500–600 cells were analyzed from 6 independent samples (*P < 0.05). Scale bar: 50 µm. **D** Flow cytometry analysis shows increased proportion of CMs overexpressing IGF2BP3 at the S and G2/M phases of the cell cycle. **E** Effects of IGF2BP3 knockdown on CM proliferation. Representative images and statistical analyses of P1 CMs stained by indicated cell proliferation markers. For each condition, 500–600 cells were analyzed from 8 independent samples (*, *P* < 0.05). Scale bar: 50 µm. **F** Flow cytometry analysis shows decreased proportion of P1 CMs at the S and G2/M phases of the cell cycle following IGF2BP3 knockdown. **G**–**I** TUNEL assay shows reduced apoptosis following overexpression of IGF2BP3 in MI heart. Data are the mean ± s.e.m. from three independent experiments (**P < 0.01). Scale bar: 50 µm.

To examine how IGF2BP3 regulates cardiac regeneration, we first performed overexpression experiments in P7 CMs because they displayed strongly reduced IGF2BP3 level in comparison with P1 CMs. Flow cytometry analysis indicated that overexpression of IGF2BP3 in P7 CMs significantly increased the proportion of phosphorylated histone H3 (pH3)-positive cells (Fig. 2A). Western blot analysis also showed an increased expression of pH3 and Aurora B proteins (Fig. 2B). These results suggest that IGF2BP3 was able to induce cell proliferation in P7 CMs. In addition, EdU incorporation and immunostaining of pH3 and Ki67 confirmed the activity of IGF2BP3 to induce mitosis in P7 CMs (Fig. 2C). Furthermore, we found that there was an increased proportion of P7 CMs overexpressing IGF2BP3 at the S and G2/M phases of the cell cycle (Fig. 2D and Supplementary Fig. S2A), indicating that IGF2BP3 was able to promote cell cycle progression. Accordingly, there was an increase in cell proliferation-related proteins including CCND1, CCNA2, and CDK2 (Supplementary Fig. S2B). Conversely, since P1 CMs showed high level of IGF2BP3 expression, we performed knockdown experiments in these cells using a previously reported small interference RNA against IGF2BP3 [25]. After transfection of si-IGF2BP3 or a non-targeting control siRNA (si-Ctrl), the knockdown efficiency was confirmed by western blot and RT-qPCR analyses (Supplementary Fig. S3). The results indicated that knockdown of IGF2BP3 significantly reduced the proportion of proliferating CMs, as revealed by EdU, pH3 and Ki67 staining (Fig. 2E). Consistently, there was a decreased proportion of CMs at the S and G2/M phases of the cell cycle (Fig. 2F and Supplementary Fig. S2C), further indicating that IGF2BP3 was involved in cell proliferation. Using an in vitro model of cardiac injury, we found that IGF2BP3 was able to protect the survival of CMs under stressed conditions, because its overexpression reduced H_2_0_2_-induced CM injury by preventing the increase of reactive oxygen species (ROS) and by increasing CM viability (Supplementary Fig. S4A, B). Moreover, overexpression of IGF2BP3 in MI heart inhibited acute inflammatory response by preventing the increase of TNFα, IL-1ß, and IL-6 (Supplementary Fig. S4C). It also reduced apoptosis of CMs after MI as determined by TUNEL assay (Fig. 2G–I). These observations strongly suggest that IGF2BP3 promotes CM proliferation and survival.

### IGF2BP3 promotes adult cardiac regeneration and improves cardiac function after myocardial infarction

**Fig. 3:**
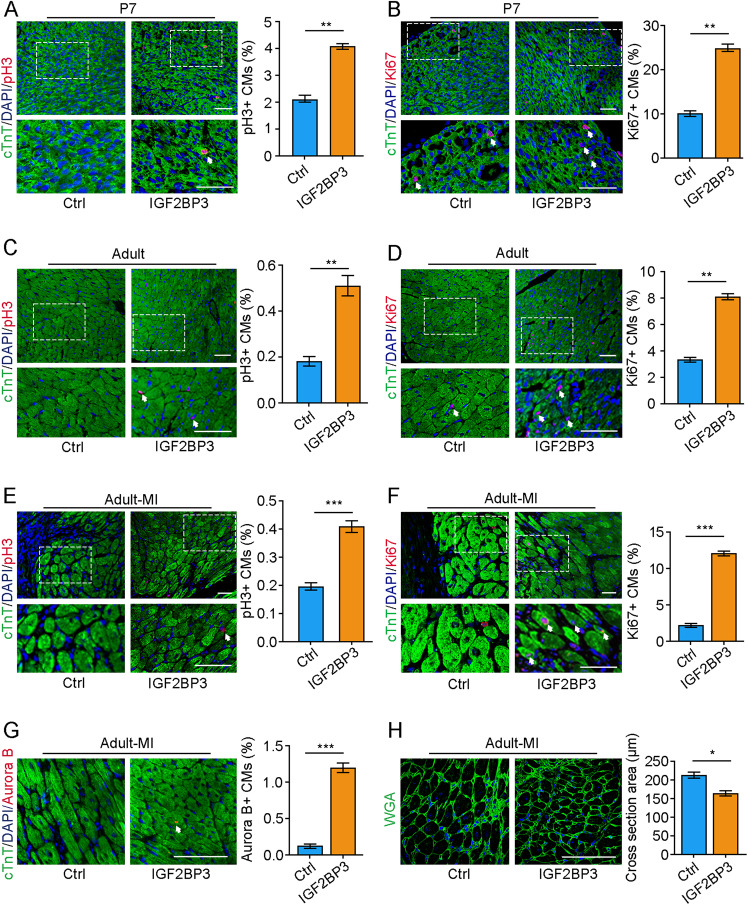
IGF2BP3 promotes cardiac regeneration in adult mice after MI. **A**–**D** Immunostaining of CMs by pH3 and Ki67 at 7 days and 56 days (adult) after overexpression of IGF2BP3 in P1 mice. Sections are also stained with cTnT to show CMs, and DAPI staining helps to count individual CMs. For each condition, 2000–3000 cells from 8 mice were included in statistical analyses (**P < 0.01). Images at the bottom of each panel are higher magnifications corresponding to the boxed regions. Scale bars: 50 µm. **E**–**G** Overexpression of IGF2BP3 increases the proportion of proliferating CMs, as shown by immunostaining of pH3 (**E**), Ki67 (**F**), and Aurora B (**G**) at 14 days post-MI. For each condition, 2100–2800 cells from 8 mice were included in statistical analyses (***P < 0.001). Images at the bottom of each panel are higher magnifications corresponding to the boxed regions. Scale bars: 50 µm. **H** Wheat germ agglutinin (WGA) staining shows increased CMs following IGF2BP3 expression in the adult MI heart, as determined by measuring the average cross section area of each CM. Data represent the mean ± s.e.m. from six independent sections using a total of 300 cells (*P < 0.05). Scale bar: 50 µm.

We next examined whether IGF2BP3 could regulate CM proliferation and cardiac regeneration in vivo. For this purpose, adeno-associated virus 9 (AAV9)-packaged IGF2BP3 was injected into the myocardium of the left ventricle of P1 mice followed by immediate MI. AAV9-packaged GFP was used to control the successful targeting (Supplementary Fig. S5A). At 7 and 56 (adult) days after injection of IGF2BP3, CM proliferation was analyzed using different markers. We found that there was a markedly increased ratio of proliferative CMs in P7 and adult mice compared with groups injected with the control vector (Ctrl), as determined by immunostaining of pH3 and Ki67 (Fig. 3A–D). HE staining and echocardiography of the adult heart showed that AAV9-mediated overexpression of IGF2BP3 did not affect the morphology of the myocardium and cardiac functions (Supplementary Fig. S5B, C). Thus, we set to analyze the effects of IGF2BP3 overexpression on myocardial repair and cardiac function in adult mice. Control vector or AAV9-packaged IGF2BP3 was injected around the peri-infarcted area of the heart, which was then subjected to MI. Interestingly, overexpression of the IGF2BP3 significantly increased the proportion of proliferative CMs in the marginal zone of the MI heart at 14 days (Fig. 3E–G). As a result, there was a reduced area of a single CM in cross section, reflecting an improvement of CM hypertrophy (Fig. 3H).

**Fig. 4:**
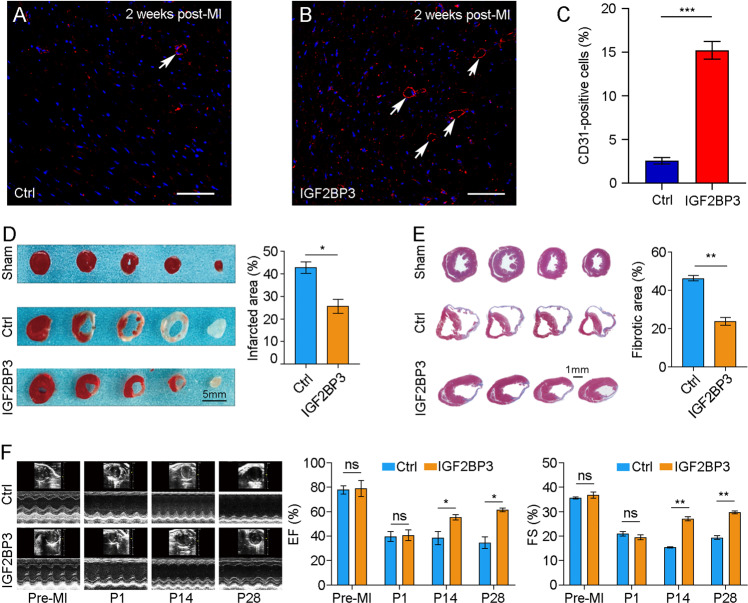
IGF2BP3 promotes myocardial regeneration and improves cardiac function after MI in adult mice. **A**–**C** Immunostaining of CD31 shows increased vasculature (arrows) in the MI heart following overexpression of IGF2BP3. Data are the mean ± s.e.m. from three independent experiments (***P < 0.001). Scale bar: 50 µm. **D** TTC staining of adult ventricular sections at 28 days post-MI shows reduced infracted area (red) following IGF2BP3 overexpression. Statistical analysis of infarcted area (white) was performed using 3 independent mice for each condition (*P < 0.05). **E** Representative images of Masson trichrome-stained adult heart sections show reduced fibrotic area at 28 days post-MI. Statistical analysis of fibrotic area was performed using 3 independent mice for each condition (**P < 0.01). **F** IGF2BP3 improves post-MI cardiac functionality. Cardiac ultrasound and analysis of ejection fraction (EF) and fractional shortening (FS) at indicated time point. Data are the mean ± s.e.m. from 3 independent mice (*P < 0.05; **P < 0.01; ns not significant).

Since restoration of vasculature can promote heart recovery after injury, we performed immunostaining of the endothelial marker CD31 on heart sections two weeks after MI. It was clear that IGF2BP3 promoted vacularization in the MI heart, as judged by an increased proportion of CD31-positive cells (Fig. 4A–C). Triphenyltetrazolium chloride (TTC) staining indicated that IGF2BP3 significantly reduced myocardial infarction size, as determined by the increased red-stained area (Fig. 4D), while Masson trichrome staining showed that it significantly reduced myocardial fibrosis (Fig. 4E). Echocardiography detected significantly preserved ejection fraction and fractional shortening (Fig. 4F). In addition, IGF2BP3 also improved the survival probability of adult mice subjected to MI (Supplementary Fig. S6). Altogether, these observations suggest that increased expression of IGF2BP3 after MI promotes adult cardiac regeneration and improves cardiac function.

### IGF2BP3-regulated gene expression in neonatal mouse cardiomyocytes

**Fig. 5:**
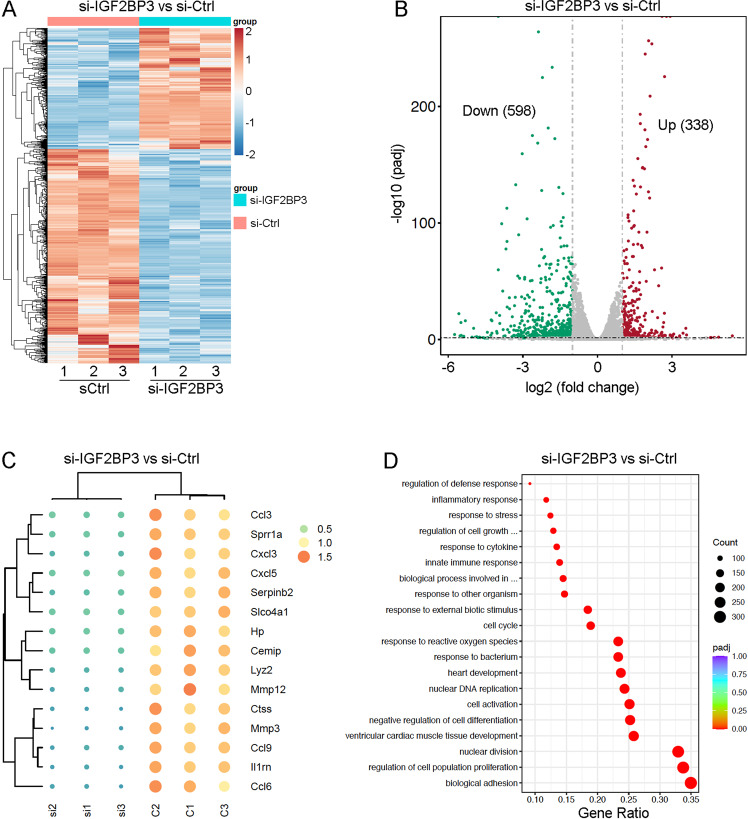
RNA-seq analysis of IGF2BP3-dependent gene expression changes in P1 CMs. **A** Heat map shows differentially expressed genes (DEGs) following IGF2BP3 knockdown (red, upregulated; blue, downregulated). **B** Volcano plot shows the numbers of differentially expressed genes. **C** Heat map shows the 15 top downregulated genes after knockdown of IGF2BP3. **D** GO analysis of the DEGs.

To investigate how IGF2BP3 regulates gene expression and identify its possible target mRNAs, we compared the transcriptomes of control (si-Ctrl) and IGF2BP3-knockdown P1 CMs using three independent samples (RNA-seq data are deposited to BioProject with accession code PRJNA954181). Analyses of heat maps and volcano plots revealed a total of 936 differentially expressed genes (DEGs), among which 338 were upregulated and 598 were downregulated in IGF2BP3-knockdown CMs (Fig. 5A, B). By unsupervised hierarchical clustering, we identified 15 most downregulated genes, including *MMP3*, *Ccl3*, *Sprr1a*, and *Cxcl3* (Fig. 5C). The observation that many chemokines were dysregulated in IGF2BP3-knockdown CMs is consistent with the important roles of chemokines in modulating cardiac fibrosis [26]. GO analysis filtered multiple terms associated with biological adhesion, regulation of cell cycle, cell proliferation, nuclear division, and ventricular cardiac muscle tissue development (Fig. 5D). GSEA analysis showed that DEGs are mainly involved in the regulation of proliferation, apoptosis, extracellular matrix (ECM) organization, and activation of matrix metalloproteinases (Supplementary Fig. S7). This analysis further confirms the above observations that IGF2BP3 promotes proliferation and vascularization for cardiac regeneration.

### Identification of *MMP3* mRNA as a target of IGF2BP3-mediated post-transcriptional regulation

**Fig. 6:**
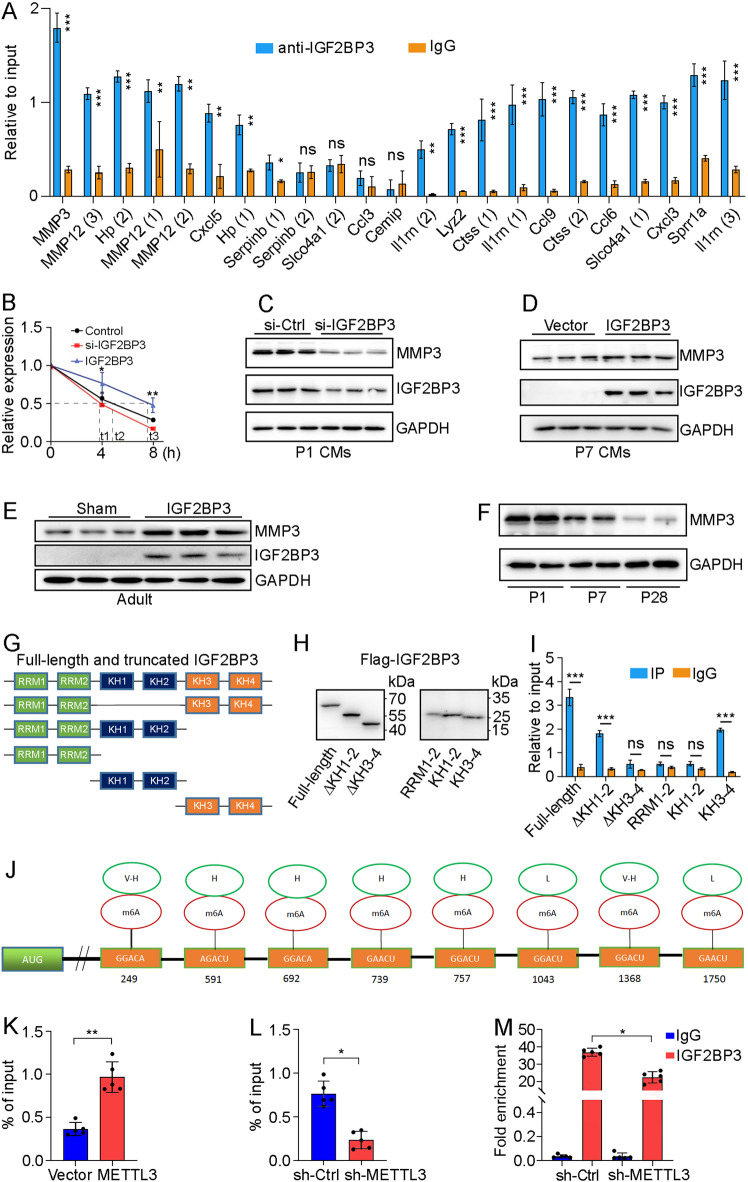
IGF2BP3 binds to *MMP3* mRNA through m6A modification. **A** RIP-qPCR analysis of IGF2BP3 interaction with indicated transcripts. Data are the mean ± s.e.m. from three independent experiments (*P < 0.05; **P < 0.01; ***P < 0.001; ns, not significant). **B** RT-qPCR analysis of *MMP3* mRNA stability following overexpression or knockdown of IGF2BP3 in actinomycin D-treated P3 CMs. Data are the mean±s.e.m. from 3 independent experiments (*P < 0.05; **P < 0.01). **C** Knockdown of IGF2BP3 decreases the expression of MMP3 protein in P1 CMs. **D** Overexpression of IGF2BP3 increases the expression of MMP3 protein in P7 CMs. **E** Overexpression of IGF2BP3 in the apical region of adult mouse heart leads to increased expression of MMP3 protein. **F** MMP3 protein expression progressively decreases during postnatal development. **G**, **H** Schematic representation and western blot analysis of Flag-tagged full-length and truncated versions of IGF2BP3. **I** RIP-qPCR analysis shows the interaction between IGF2BP3 KH3 and KH4 domains with *MMP3* mRNA. Data are the mean ± s.e.m. from three independent experiments (***P < 0.001; ns, not significant). **J** Predicted m6A recognition sites in *MMP3* mRNA with indicated confidence levels (V-H, very high; H, high; L, low). **K**, **L** MeRIP enrichment followed by RT-qPCR analysis shows METTL3-dependent *MMP3* mRNA methylation modification. Data are the mean±s.e.m. from 5 independent experiments (*P < 0.05; **P < 0.01). **M** RIP-qPCR analysis shows that knockdown of METTL3 reduces the binding of IGF2BP3 to *MMP3* mRNA. Data are the mean ± s.e.m. from five independent experiments (*P < 0.05).

Since IGF2BP3 post-transcriptionally regulates gene expression through binding to its target mRNAs, we further validated the RNA-seq data by RNA immunoprecipitation (RIP)-qPCR assay of the 15 most downregulated genes represented by 23 transcripts (Fig. 6A). Among several mostly enriched mRNAs by IGF2BP3 antibody, we chose *MMP3* for further analysis because its function in cardiac regeneration has not been reported to date. To understand how IGF2BP3 regulates MMP3 expression in CMs, we first examined the stability of *MMP3* mRNA following knockdown or overexpression of IGF2BP3 in the presence of actinomycin D to prevent de novo transcription. Clearly, knockdown of IGF2BP3 decreased, whereas expression of IGF2BP3 increased the half-life of *MMP3* mRNA in P3 CMs (Fig. 6B). Accordingly, knockdown of IGF2BP3 in P1 CMs reduced MMP3 protein level, whereas overexpression of IGF2BP3 in P7 CMs produced the opposite effect (Fig. 6C, D). Moreover, injection of IGF2BP3 in the apical myocardial region of adult mice also led to increased expression of MMP3 protein (Fig. 6E). Interestingly, MMP3 protein displays a similar expression profile as IGF2BP3 in the heart, with progressively decreased protein levels during postnatal development (Fig. 6F). These results indicate that IGF2BP3 plays a role in the post-transcriptional regulation of MMP3 expression in CMs.

To provide mechanistic insights into the biochemical interaction between IGF2BP3 and *MMP3* mRNA, we generated truncated IGF2BP3 variants by deleting different functional domains and examined their ability to interact with *MMP3* mRNA by RIP-qPCR. IGF2BP3 comprises two RNA recognition motifs (RRMs) at the N-terminal region and four hnRNP K homology (KH) domains located at the central and C-terminal regions (Fig. 6G). Western blot analysis indicated that Flag-tagged full-length IGF2BP3 and truncated variants were appropriately expressed in CMs (Fig. 6H). We found that only the last two KH domains (KH3-4) were able to interact with *MMP3* mRNA. Although KH1-2 did not bind to *MMP3* mRNA, its absence seemed to reduce the binding of IGF2BP3 to *MMP3* mRNA (Fig. 6I). This suggests that IGF2BP3 binds to *MMP3* mRNA through its KH3 and KH4 domains, which is probably assisted by the KH1 and KH2 domains. We further analyzed the sequence in *MMP3* mRNA responsible for interaction with IGF2BP3. Since IGF2BP3 mostly functions as a m6A reader and recognizes the GG(m6A)C consensus sequence in target mRNAs [21], there is a possibility that it also interacts with *MMP3* mRNA through this type of RNA modification. Indeed, the sequence-based RNA adenosine methylation site predictor (SRAMP) program (http://www.cuilab.cn/sramp) identified several high-confidence methylation modification sites on the *MMP3* mRNA (Fig. 6J). MeRIP enrichment experiments using anti-m6A antibody followed by RT-qPCR analysis indicated that *MMP3* mRNA was subjected to m6A modification, which is dependent on METTL3 function (Fig. 6K, L). Importantly, RIP-qPCR assay showed that knockdown of METTL3 using short hairpin RNAs (shRNAs) targeting its mRNA significantly reduced the binding of IGF2BP3 with *MMP3* mRNA (Fig. 6M and Supplementary Table S3). This is consistent with the function of IGF2BP3 as a methylation reader protein specific to m6A and suggests that it interacts with *MMP3* mRNA in an m6A-dependent manner. However, we cannot exclude the possibility that IGF2BP3 may also interact with other motifs present in *MMP3* mRNA. Altogether, these results clearly show that IGF2BP3 regulates MMP3 protein expression by directly binding to and stabilizing its mRNA through m6A modification.

### IGF2BP3 promotes CM proliferation via MMP3

**Fig. 7:**
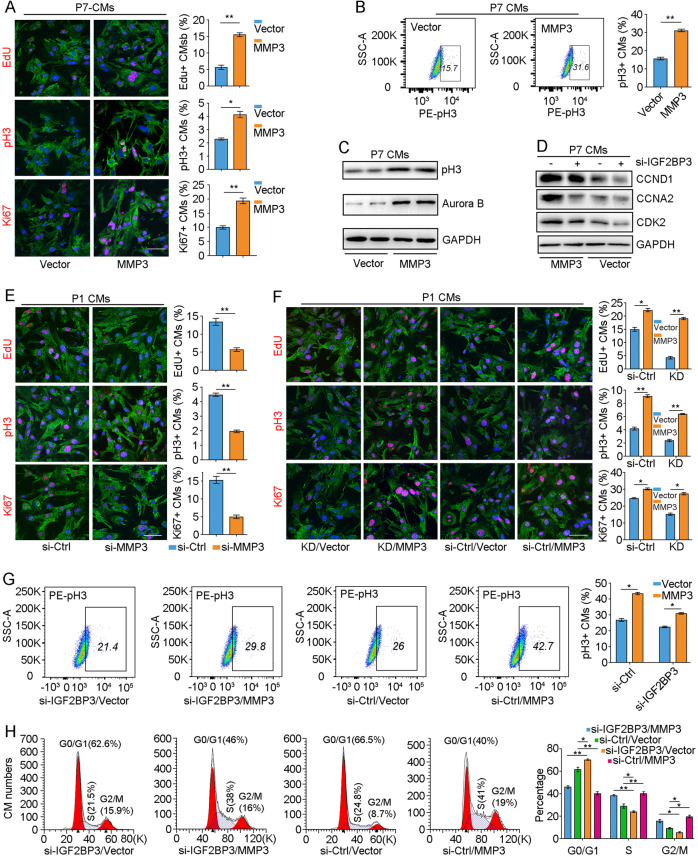
MMP3 functions downstream of IGF2BP3 in CM proliferation. **A**–**C** Overexpression of MMP3 in P7 CMs promotes cell proliferation, as revealed by flurescence staining (**A**), flow cytometry sorting (**B**), and western blot analysis (**C**) of cell cycle-related proteins. Statistical analyses of proliferating cells stained by EdU, pH3, and Ki67 were performed using 500–600 cells from 6 independent samples in each condition (*P < 0.05; **P < 0.01). Scales bar: 50 µm. **D** Western blot analysis shows that overexpression of MMP3 rescues the inhibitory effects of IGF2BP3 knockdown on the expression of cell cycle-related proteins. **E** Knockdown of MMP3 in P1 CMs decreases cell proliferation. Scale bar: 50 µm. For each condition, about 600 cells from six independent samples were included in statistical analyses (**P < 0.01). **F** Overexpression of MMP3 rescues the inhibitory effects of IGF2BP3 knockdown (designated as KD) on P1 CM proliferation. Scale bar: 50 µm. For each condition, about 600–900 cells from six independent samples were included in statistical analyses (*P < 0.05; **P < 0.01). **G** Flow cytometry analysis shows that overexpression MMP3 rescues CM proliferation inhibited by IGF2BP3 knockdown (*P < 0.05). **H** Flow cytometry analysis shows rescue effects of MMP3 overexpression on cell cycle progression inhibited by IGF2BP3 knockdown (*P < 0.05; **P < 0.01).

Having identified *MMP3* mRNA as a post-transcriptional target of IGF2BP3 in CMs, we wondered how the two genes functionally interact in CM proliferation. First, the effects of MMP3 overexpression or knockdown on CM proliferation were examined using EdU labeling as well as pH3 and Ki67 immunostaining. As observed with IGF2BP3, overexpression of MMP3 also promoted proliferation of P7 CMs (Fig. 7A). This was further confirmed by flow cytometry analysis of pH3-positve CMs and by western blot analysis of pH3 and Aurora B proteins (Fig. 7B, C).

**Fig. 8:**
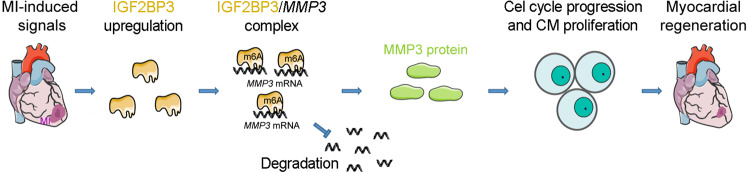
Model of functional interaction between IGF2BP3 and MMP3 in cardiac regeneration. MI-induced signals activate IGF2BP3 expression in CMs. IGF2BP3 in turn stabilizes *MMP3* mRNA by interacting with m6A methylation modification through the KH3 and KH4 domains. MMP3 may be implicated in remodeling the ECM to promote CM proliferation and regeneration.

We next examined the functional interaction of IGF2BP3 and MMP3 in CM proliferation by rescue experiments. In P7 CMs, overexpression of MMP3 was able to rescue the expression levels of cell cycle-related proteins, including CCND1, CCNA2 and CDK2, caused by IGF2BP3 knockdown (Fig. 7D). We then designed 3 siRNAs against MMP3 and found that one of them (si-MMP3-810) was more potent to inhibit MMP3 expression (Supplementary Fig. 8). In P1 CMs, knockdown of MMP3 significantly inhibited proliferation (Fig. 7E), whereas overexpression of MMP3 reversed the effects of IGF2BP3 knockdown (KD) on CM proliferation (Fig. 7F, G), as shown by EdU staining, pH3 and Ki67 immunostaining, and flow cytometry analysis of pH3-positive cells. This rescuing effect was further confirmed by the increased ratio of CMs at S and G2/M phases of the cell cycle (Fig. 7H). Therefore, these results strongly demonstrate that MMP3 represents a novel target and functions downstream of IGF2BP3 to promote cell proliferation. Mechanistically, the KH3 and KH4 domains of IGF2BP3 bind to and stabilize *MMP3* mRNA to promote its translation, which may be enhanced by the KH1 and KH2 domains. As a metalloproteinase implicated in the breakdown of ECM, tissue remodeling, and wound repair, increased expression of MMP3 will likely promote proliferation of CMs and cardiac regeneration after MI, thus improving cardiac function (Fig. 8).

## Discussion

Persistent loss of CMs following MI can lead to heart failure. The adult mammalian heart only displays a modest regenerative capacity, whereas the heart from early postnatal mice and from adults of lower vertebrates can achieve complete regeneration after ventricular resection by inducing the proliferation of existing CMs [27**–**29]. Therefore, understanding the molecular mechanism underlying reactivation of cell proliferation is essential to induce cardiac regeneration after myocardial injury in adults. In this study, we have demonstrated that IGF2BP3 expression was progressively downregulated during postnatal mouse development but was rapidly upregulated in CMs following MI. It plays an important role in the post-transcriptional regulation of cardiac regeneration. Mechanistically, IGF2BP3 binds to and stabilizes *MMP3* mRNA through m6A methylation modification, leading to increased expression of MMP3 protein. Functional analyses suggest that MMP3 acts downstream of IGF2BP3 to promote CM proliferation. Our findings identify *MMP3* mRNA as a novel target of IGF2BP3 and provide insights into the molecular frameworks contributing to heart regeneration.

IGF2BP3 is one of the three members of the highly conserved IGF2BP family. It regulates a wide range of physiological and pathological processes, particularly during tumorigenesis and tumor progression [30]. As a RNA-binding protein, IGF2BP3 is involved in the post-transcriptional regulation of gene expression at multiple levels. However, the mechanisms by which it regulates cardiac regeneration are incompletely understood. Recently, it has been shown that IGF2BP3 can enhance neonatal heart regeneration in mice [23]. We further extended previous works by showing that the expression of IGF2BP3 is rapidly upregulated following MI and that overexpression of IGF2BP3 can also promote CM proliferation and cardiac regeneration in adult mice. It is worth mentioning that after MI, the upregulation of IGF2BP3 in the adult heart is weak compared with the neonatal heart. This implies that the limited regenerative capability of the adult mammalian heart may be at least partially due to the inability to fully reactivate the IGF2BP3-related signaling networks. It raises a possibility that manipulation of IGF2BP3 expression level could contribute to improve mammalian heart regeneration in the adult.

Our RNA-seq analyses revealed that *MMP3* mRNA is one of the targets of IGF2BP3 in CMs. It has been shown recently that IGF2BP3 functions as an m6A reader to promote mRNA stability and translation [22]. Consistent with this observation, we found that IGF2BP3 binds to *MMP3* mRNA in an m6A-dependent manner. This suggests that m6A reader and writer proteins may play important roles in CM proliferation. They are either positively or negatively associated with cardiac regeneration in a target-dependent manner. Indeed, evidence is accumulating that m6A modifications play essential roles in cardiovascular diseases [31]. Knockout of the *METTL3* gene in mice triggers re-entry of CMs into the cell cycle and decreases infarct size after MI by inhibiting miR-143 m6A modification [32]. This is consistent with our observation that methyltransferases METTL3 and METTL14 display increased expression during postnatal development. ALKBH5 regulates CM proliferation and heart regeneration by demethylating *YTHDF1* mRNA [33]. Overexpression of FTO in a mouse MI model reduces cardiac fibrosis and enhances cardiac angiogenesis by selectively demethylating cardiac contractile transcripts and preventing their degradation [34]. In this work, we found that m6A methylation modification of *MMP3* mRNA by METTL3 may be important for cardiac regeneration. Thus, METTL3-mediated m6A modification should exert complex roles in cardiac development and regeneration, likely dependent on the target mRNAs.

MMP3 is a member of the MMP family that functions to degrade components of the ECM for tissue remodeling [35]. Accumulating evidence indicate that MMP proteins, including MMP3 (also known as stromelysin-1), are closely associated with MI and heart failure [36**–**39]. Thus they can be used as biomarkers and potential therapeutic targets for cardiovascular diseases [40**–**42]. Moreover, polymorphisms of the *MMP3* gene are correlated with the genetic risk of MI, suggesting a possible contribution of this gene to the occurrence of this condition [43, 44]. The mechanisms underlying MMP3 function in cardiac regeneration remain elusive. It is likely that increased expression of MMPs contributes to changes of the ECM and post-MI remodeling of CMs [36, 45]. This is consistent with the essential roles of ECM in heart development and repair [46]. MMPs can also function to initiate cellular signaling cascades to promote would repair, thereby contributing to cell proliferation [45]. Indeed, there is evidence that different MMPs can regulate vascular cell proliferation and vascular remodeling through proteolytic cleavage and activation of bioactive molecules such as inflammatory cytokines [47]. At least in the porcine endometrium, MMP3 has been shown to promote endothelial cell proliferation and capillary formation [48]. Therefore, controlled activity of MMPs is crucial for heart development and regeneration. Since MMPs generally function together with tissue metalloproteinase inhibitors (TIMPs) to regulate ECM, a mis-balance between synthesis and degradation will disrupt ECM and tissue homeostasis [49]. We have provided evidence that IGF2BP3 regulates MMP3 expression for CM proliferation, raising the possibility they may function together in cardiac regeneration after MI.

IGF2BP3 may also promote cardiac regeneration independently of MMP3. It has been shown that IGF2 plays a role in promoting CM proliferation during heart development [50, 51]. Therefore, IGF2BP3 may enhance IGF2 signaling by stabilizing *IGF2* mRNA. Because IGF2BP3 is a known regulator of proteins associated with cell proliferation and cell cycle progression [52], there is also the possibility that it promotes CM proliferation during cardiac regeneration by directly regulating the expression of these proteins. Further studies will be necessary to address these questions. It will be also of interest to determine the implication of MMP3 in cardiac regeneration by gain- and loss-of-function analyses using in vivo animal models.

## Materials and methods

### Animals

C57BL/6 mice were purchased from the Animal Center of Nanjing (Nanjing, China). All animal studies were approved by the Animal Care Committee of Guangdong Medical University and performed according to the ARRIVE guidelines.

### Plasmid constructs and adeno-associated virus 9 injection

Full-length and truncated *IGF2BP3* coding sequences were cloned in the CV702 vector (pCMV-MCS-3FLAG-SV40-Puromycin) in-frame with the 3 Flag epitopes. The coding sequence of METTL3 was also cloned in the CV702 vector, and the coding region of *MMP3* was cloned in the pcDNA vector. All constructs were verified by sequencing before use.

Mice (2-month-old) were randomly divided into sham and MI groups. They were subjected to deep anesthesia with 5% isoflurane for 2 min through tracheal intubation followed 2% isoflurane until the end of the operation. After surgical opening of the fourth intercostal space, the left coronary artery was ligated with a prolene suture (size 8-0) to induce MI. For overexpression, 1 × 10^11^ vg (viral genome)/mL purified viruses packaged with the gene of interest in 50 µL of phosphate-buffered saline (PBS) were injected into the myocardium bordering the infarct zone. Neonatal mice at 7 days were anesthetized by hypothermia on ice for 1.5 min, followed by surgical opening of the fourth intercostal space and injection of purified viruses into the left ventricular wall. After injection, they were recovered under warm temperature.

### Isolation and culture of CMs

Neonatal mice were anesthetized through isoflurane (2%) inhalation and sacrificed by cervical dislocation. Small pieces of ventricles were treated with 0.1% trypsin (Solarbio, Beijing, China) at 4 °C for 15 h. They were then digested twice (15 min each) with 0.1 mg/mL type II collagenase (Solarbio, Beijing, China) in PBS containing 5 mg/mL bovine serum albumin (Sigma, St. Louis, USA) at 37 °C under constant stirring. After each digestion, cell suspension was mixed with two volumes of DMEM medium containing 10% fetal bovine serum (Gibco, Grand Island, USA). Dissociated cells were collected by centrifugation and resuspended in DMEM medium supplemented with 10% fetal bovine serum and 1% penicillin-streptomycin (Beyotime, Beijing, China). They were cultured in a plastic dish (100 mm) for 1.5 h at 37 °C to separate cardiac fibroblasts that adhere to the culture dish. CMs that remain suspended in culture medium were collected by centrifugation. They were plated on plastic dishes coated with 0.1% gelatin and cultured at 37 °C in a humidified incubator under 5% CO_2_ atmosphere for appropriate period.

### RNA extraction and RT-qPCR

Total RNA was extracted using TRIzol reagent (Merck, Massachusetts, USA). After reverse transcription, samples were analyzed on the Roche LightCycler 480 system (Roche Applied Science, Switzerland) using the qPCR kit (TAKARA, Japan) in the presence of gene-specific primers (Supplementary Table S1). Results were calculated using the 2^-ΔΔCt^ method with GAPDH as an internal control.

### Western blot analysis

Tissues or cells were homogenized in RIPA buffer with 1 mM phenylmethylsulfonyl fluoride (PMSF). Protein concentrations were determined by enhanced bicinchoninic acid (BCA) protein assay (Beyotime, Beijing, China). Samples were mixed with loading buffer and heated for 10 min at 100 °C. Proteins were separated by 10% or 12% polyacrylamide gel and transferred onto PVDF membranes (Millipore, Massachusetts, USA). After blocking with 5% non-fat milk, the membranes were incubated at 4 °C overnight with primary antibodies followed by peroxidase-conjugated secondary antibodies (Supplementary Table S2). Protein bands were visualized using Luminata Western HRP substrates (Millipore) and Tanon 5200 chemiluminescence imaging system (Shanghai, China).

### Transfection of plasmids, siRNAs, and shRNAs

Transfection of plasmid DNA was performed using Lipofectamine 3000 (Invitrogen, Waltham, USA). For knockdown experiments, CMs were transfected with gene-specific siRNA or plasmids allowing the expression of shRNAs using Lipofectamine RNAiMAX (Invitrogen, Waltham, USA) or Lipofectamine 3000, respectively. Non-targeting siRNA or shRNAs (si-Ctrl or sh-Ctrl) were used as a specificity control (Supplementary Table S3). Cells were harvested 48 h after transfection and analyzed using appropriate methods.

### RNA sequencing

The quality of extracted RNAs was determined using NanoDrop2000 (Thermo Scientific, Waltham, USA). Samples were tested for library construction and sequenced using the Illumina Novaseq 6000 (Illumina, San Diego, USA). Sequenced reads were mapped onto the mouse reference genome (http://asia.ensembl.org/index.html). FPKM (fragments per kilobase per million mapped reads) was used to determine gene expression levels. Differentially expressed genes were analyzed using EdgeR software. KEGG and GO enrichments were used to determine their involvement in different biological processes.

### Masson staining

The hearts were fixed in 4% paraformaldehyde for 48 h at room temperature. They were then dehydrated in ethanol and xylene. Sections of 50 µm thickness were cut from paraffin-embedded tissues every 200 µm from the ligature site to the apex of the heart. Four sections were collected from each heart and stained with Masson trichrome (Merck, New Sawamoto State, USA). The area of fibrosis was measured by Image J software.

### TTC staining

Heart tissues dissected from euthanized animals were washed in PBS and immediately frozen at −80 °C for 15 min. Small tissue slices were prepared and incubated in 1% TTC (Merck, New Sawamoto State, USA) at 37 °C for 30 min. Non-infarcted area were stained in red, whereas infarcted area appeared white. Stained slices were imaged and analyzed by Image J software.

### Immunofluorescence, EdU staining, and TUNEL labeling

Primary CMs were fixed in 4% paraformaldehyde, and permeabilized using 0.5% Triton-X100 in the presence of 5% normal goat serum (Solarbio, Beijing, China). Heart tissues were frozen sectioned at 5 µm thickness using a cryostat (Leica). Samples were incubated overnight in primary antibodies (Supplementary Table S2) at 4 °C followed by several washes in PBS. After incubation in the secondary antibody, cell nuclei were stained with Hochest33342 or DAPI (Merck, New Sawamoto State, USA). DNA synthesis was monitored by EdU labeling using the Cell-Light^TM^ EdU Apollo 567 In Vitro Kit (RIBOBIO, Guangzhou, China). Images were acquired using OLYMPUS FV3000 confocal microscope (OLYMPUS, Japan). Labeling of apoptosis was performed using the TUNEL Kit (Beyotime, Beijing, China).

### MeRIP assay

This was performed using the Magna MeRIP™ m6A Kit (Millipore, Massachusetts, USA) by following the manufacture’s protocol. RNAs were chemically fragmented into smaller fragments (≤100 nucleotides) followed by magnetic immunoprecipitation using a monoclonal antibody against m6A. Isolated RNA fragments were analyzed by RT-qPCR using gene-specific primers (Supplementary Table S1).

### RNA immunoprecipitation

This was performed using Magna RIP™ RNA-Binding Protein Immunoprecipitation Kit (Millipore, Massachusetts, USA) by following the manufacture’s instruction. Cells were lysed in RIP Lysis Buffer and the lysates were incubated with antibodies against IGF2BP3 or with a negative IgG control, which were pre-coupled with magnetic beads. The bound complexes were precipitated and washed with RIP wash buffer using a magnet. Immunoprecipitated RNAs were subjected to RT-qPCR analysis using gene-specific primers (Supplementary Table S1).

### Flow cytometry analysis of cell cycle

Cells were washed with PBS and fixed overnight in 75% ethanol. They were incubated in the dark with 10 mg/mL of RNase A and 5 mg/mL of propidium iodide (Beyotime, Beijing, China) for 30 min at room temperature. The samples were analyzed using BD FACSCanto II flow cytometer (Becton, Dickinson and Company, New Jersey, USA) and the FlowJo software.

### Reactive oxygen species assay

CMs were treated with H_2_O_2_ (500 µM) to induce weak injury. Cells were then incubated with 10 mM DCFH-DA probe (Solarbio, Beijing, China) and digested with EDTA-free trypsin. After centrifugation, cells were resuspended in serum-free solution and analyzed using the BD FACSCanto II flow cytometer. The mean fluorescence intensity was used to represent ROS level.

## Supplementary information

Original Data File

Supplemental Materials

## Data Availability

Data and reagents reported in this work are available upon request to the corresponding authors.
