# Supplementary material for: IGF2BP3 promotes adult myocardial regeneration by stabilizing MMP3 mRNA through interaction with m6A modification
Source: Cell Death Discov. 2023 May 15;9:164. doi: 10.1038/s41420-023-01457-3 (PMC10185520; doi:10.1038/s41420-023-01457-3)
Supplement: Supplementary file 1 — Original Data File [file 41420_2023_1457_MOESM1_ESM.pdf]

Uncropped western blot images of the indicated Figures.

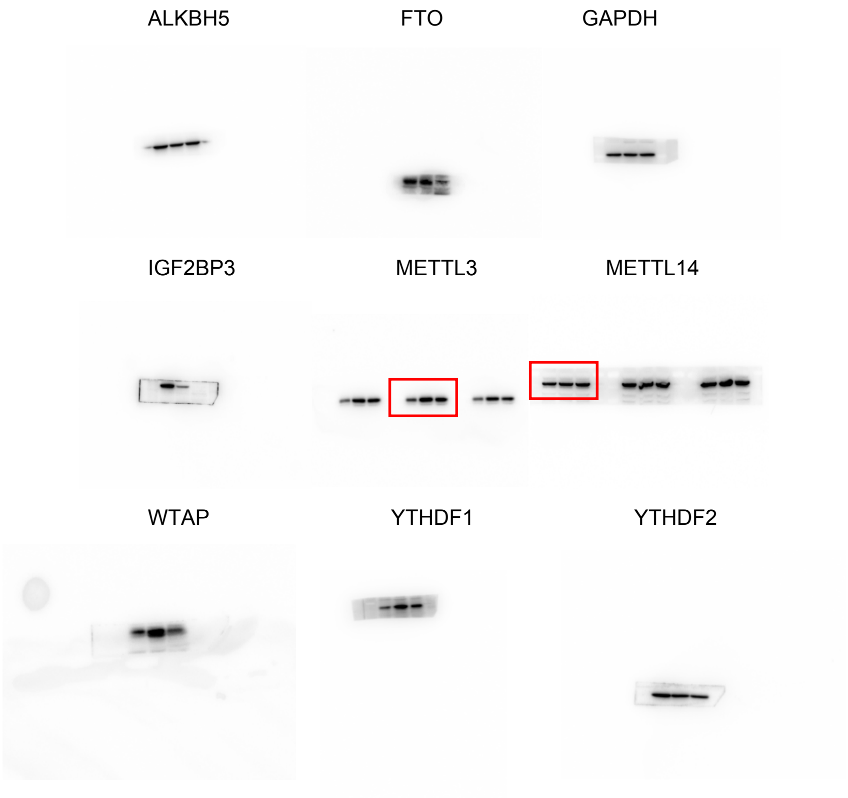


Figure 1A

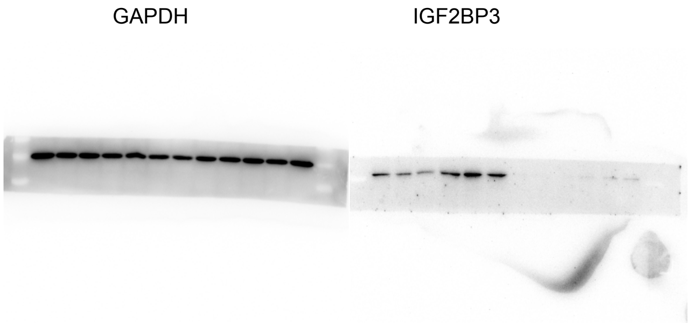


Figure 1E

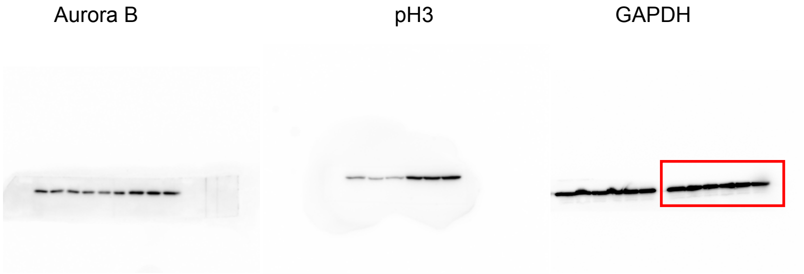


Figure 2B

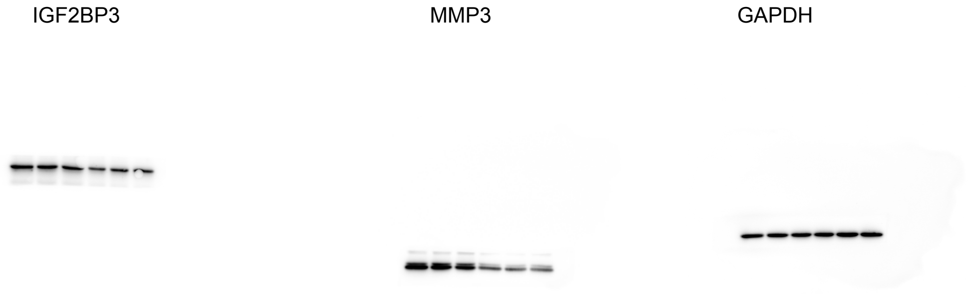


Figure 6C

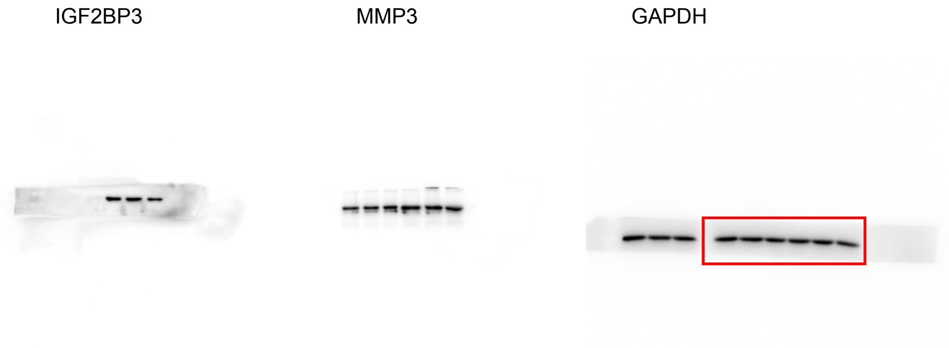


Figure 6D

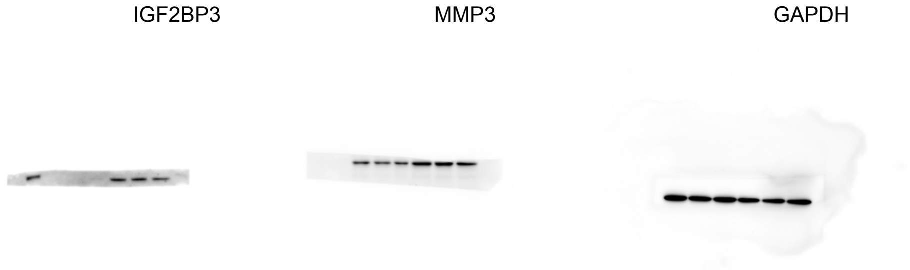


Figure 6E

MMP3 GAPDH

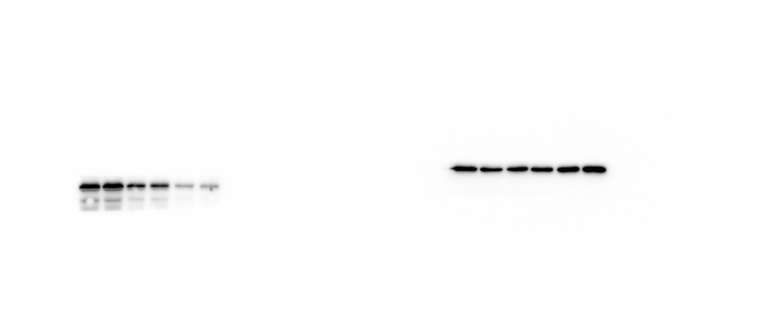

Figure6F

Flag-IGF2BP3 Flag-IGF2BP3

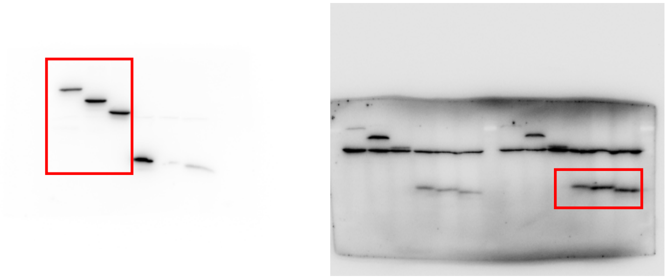

Figure 6H

Aurora B pH3 GAPDH

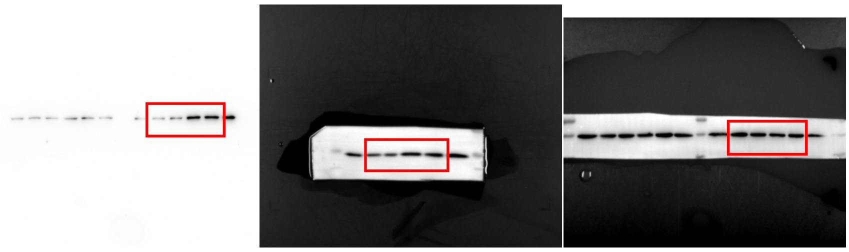

Figure 7C

CCNA2 CDK2 CCND1 GAPDH

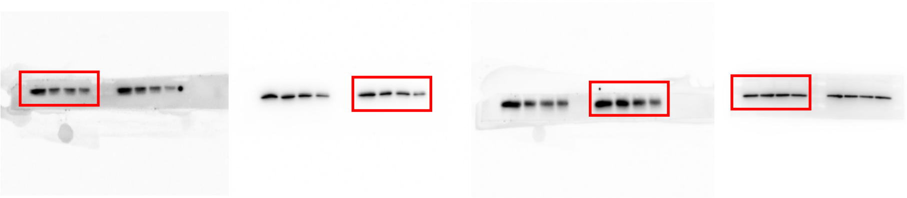

Figure 7D

IGF2BP3 GAPDH

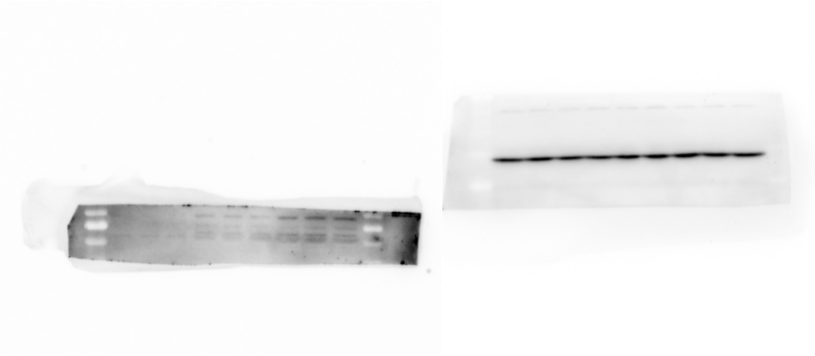

Figure S1A

IGF2BP3 GAPDH

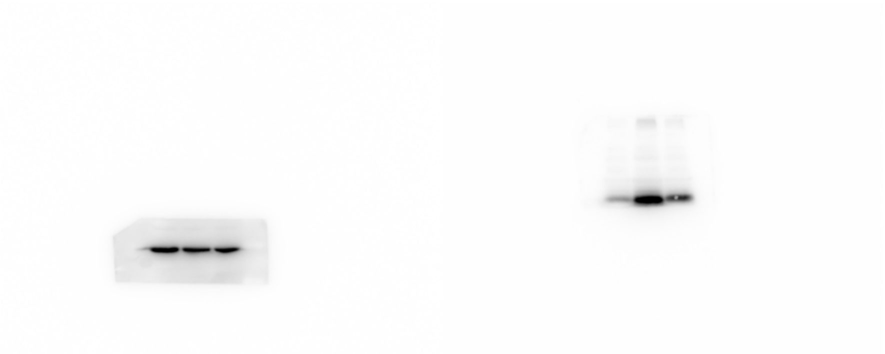

Figure S1B

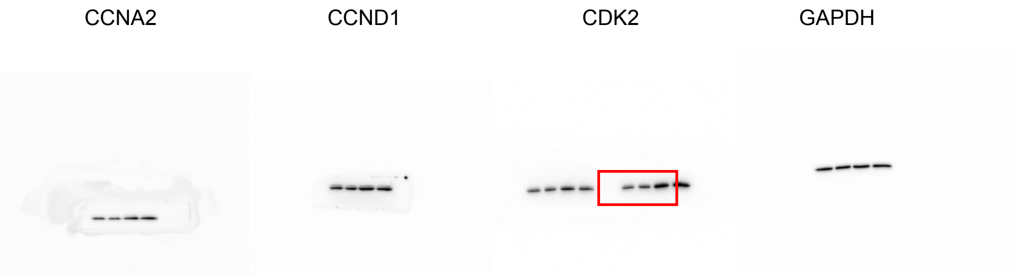


Figure S2B

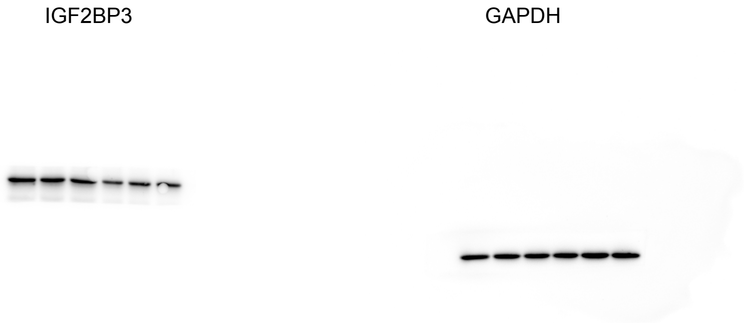


Figure S3B

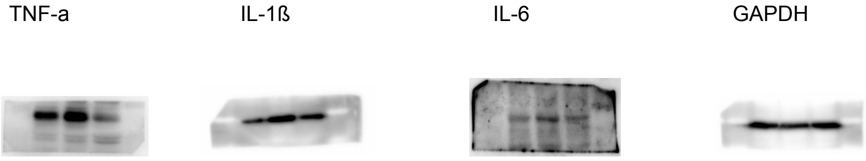


Figure S4C

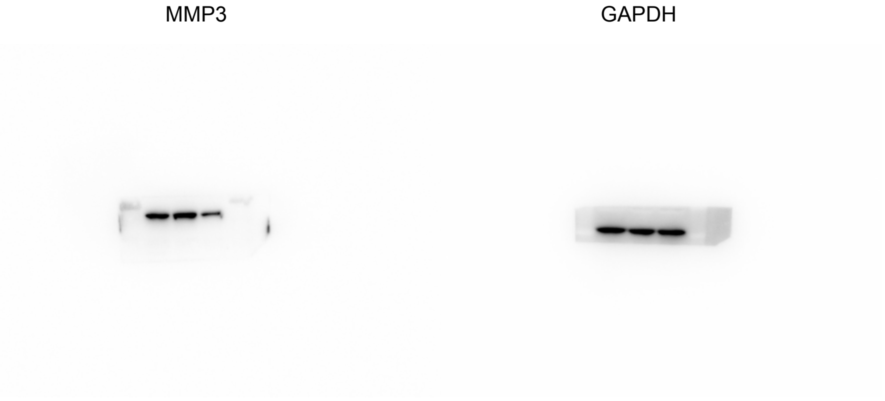


Figure S8B
