# Supplementary material for: IGF2BP3 promotes adult myocardial regeneration by stabilizing MMP3 mRNA through interaction with m6A modification
Source: Cell Death Discov. 2023 May 15;9:164. doi: 10.1038/s41420-023-01457-3 (PMC10185520; doi:10.1038/s41420-023-01457-3)
Supplement: Supplementary file 2 — Supplemental Materials [file 41420_2023_1457_MOESM2_ESM.pdf]

Supporting materials for:

IGF2BP3 promotes adult myocardial regeneration by stabilizing *MMP3* mRNA through interaction with N6-methyladenosine modification

Simeng Li, Siman Shen, Hao Xu, Shuyun Cai, Xiaodong Yuan, Changsen Wang, Xiaojun Zhang, Suyun Chen, Jianning Chen, De-Li Shi, Liangqing Zhang

Authors for correspondence: De-Li Shi and Liangqing Zhang

This PDF file includes:

Figures S1 to S8

Tables S1 to S3

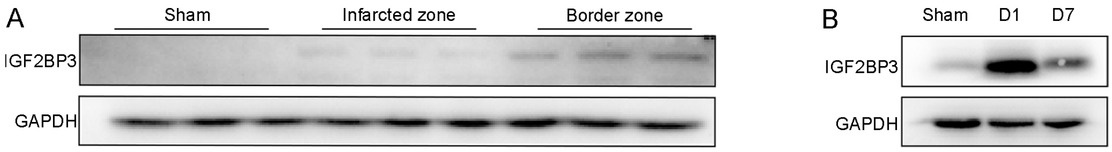

Figure S1. IGF2BP3 expression in MI heart. (A) Increased expression of IGF2BP3 in the border zone of adult heart at day 7 post-MI. (B) Expression of IGF2BP3 at day 1 and 7 post-MI in adult mice.

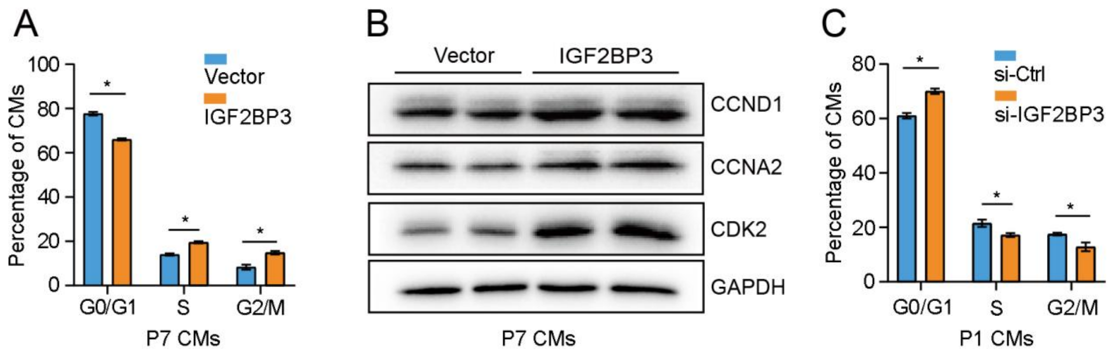

Figure S2. Regulation of cell cycle progression in CMs by IGF2BP3. (A) Flow cytometry analysis of IGF2BP3-overexpressing P7 CMs at different phases of the cell cycle (*, $P < 0.05$). (B) IGF2BP3 increases the expression of proteins associated with cell cycle progression in P7 CMs. (C) Flow cytometry analysis shows the inhibitory effects of IGF2BP3 knockdown on cell cycle progression in P1 CMs (*, $P < 0.05$).

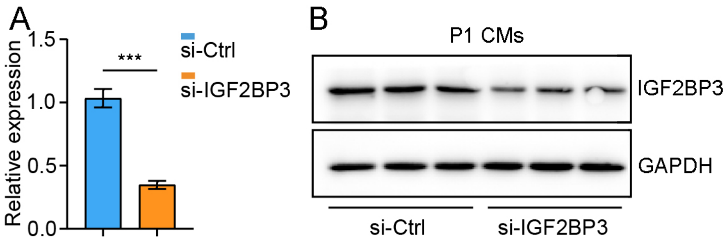

Figure S3. Efficiency of IGF2BP3 knockdown. (A) RT-qPCR analysis of *IGF2BP3* mRNA levels in P1 CMs. Values in the si-Ctrl condition are set to 1 as a reference, after normalization to GAPDH. Data are the mean±s.e.m. from three independent samples (***, $P < 0.001$). (B) Western blot analysis of IGF2BP3 expression following its knockdown in P1 CMs.

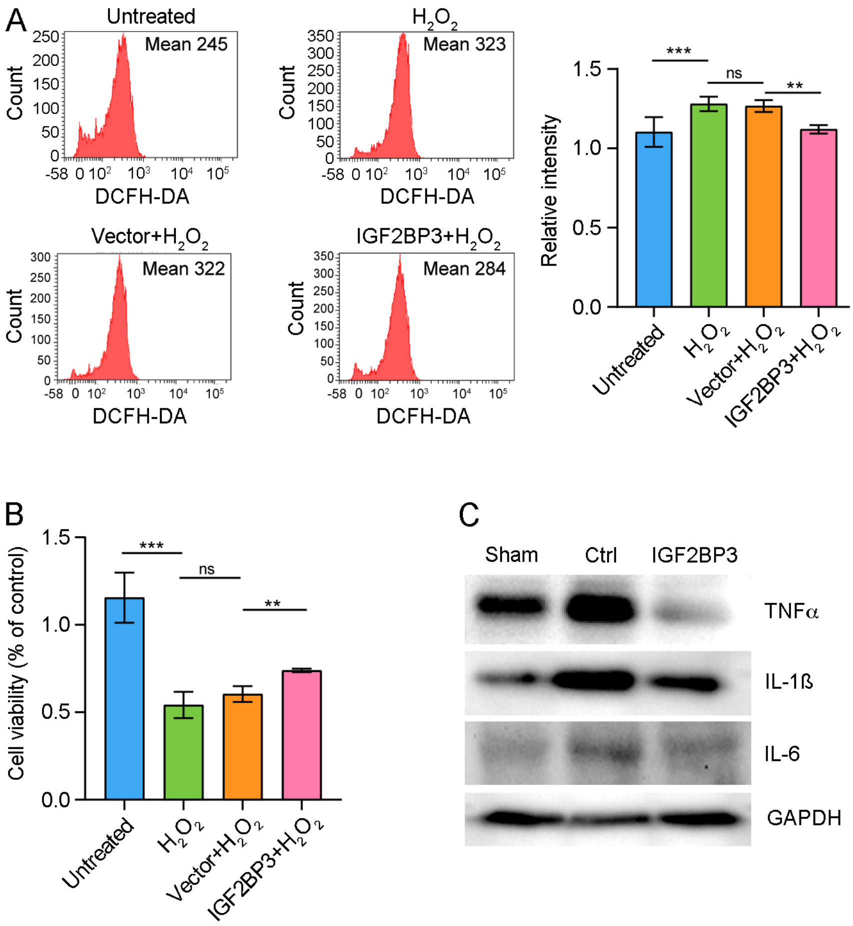

Figure S4. Protection of CM damage by IGF2BP3. (A) Measurement and statistical analysis of ROS levels in indicated conditions following treatment with H_2O_2 (**, $P < 0.01$; ***, $P < 0.001$; ns, not significant). (B) CCK-8 cell proliferation and cytotoxicity assay shows the protective effect of IGF2BP3 on cell viability (**, $P < 0.01$; ***, $P < 0.001$; ns, not significant). (C) Western blot analysis of TNFα, IL-1ß and IL-6 protein levels shows reduced acute inflammatory response following IGF2BP3 overexpression in adult heart 2 days post-MI.

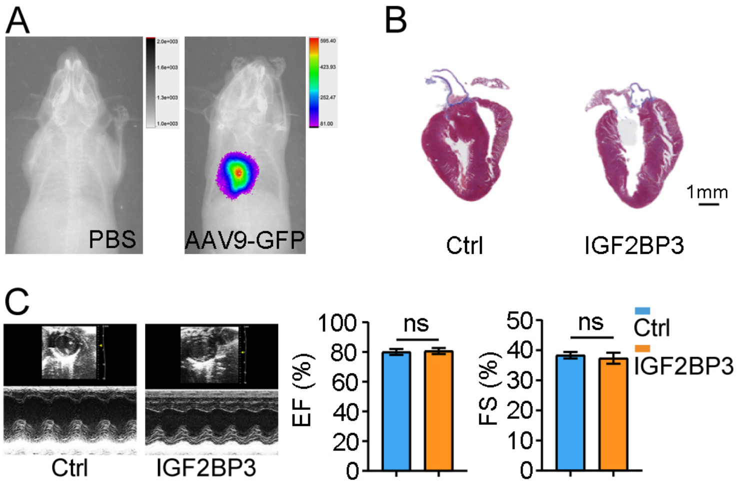

Figure S5. Overexpression of IGF2BP3 does not affect heart morphology and function. (A) Verification of successful targeting of apical myocardial region using AAV9-packaged GFP as a control. (B) HE staining of adult mouse hearts shows comparable morphology between control and IGF2BP3-overexpressing conditions. (C) Adult mouse cardiac ultrasound in control and IGF2BP3-overexpressing conditions shows similar function (ns, not significant).

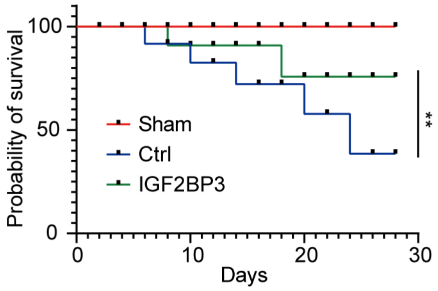

Figure S6. IGF2BP3 protects survival of adult MI mice. Kaplan-Meier survival curve shows increased survival rate of adult MI mice following injection of IGF2BP3 in the heart (n = 30 mice for each group; **, P < 0.01).

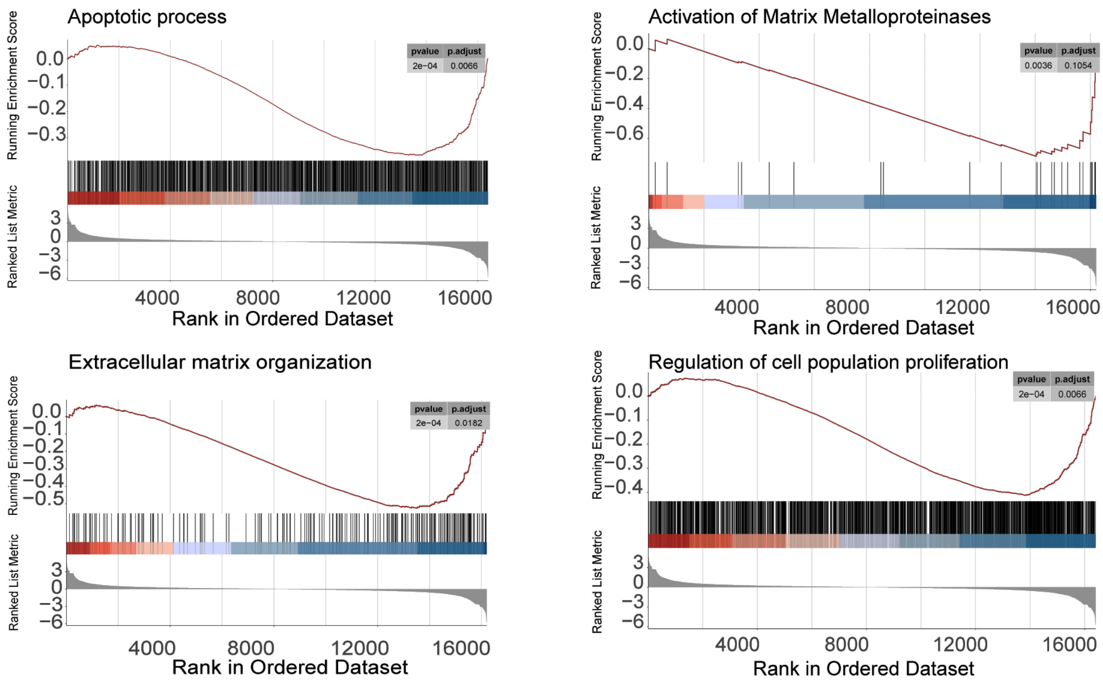

Figure S7. IGF2BP3-regulated biological processes in P1 CMs. Gene set enrichment analysis (GSEA) analysis of the DEGs identified by RNA-seq.

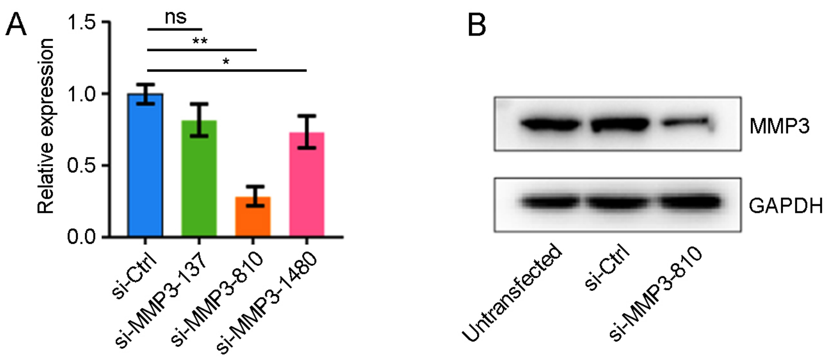

Figure S8. Efficiency of *MMP3* knockdown. (A) RT-qPCR analysis of *MMP3* expression shows the efficiency of the 3 siRNAs against MMP3 (*, $P < 0.05$; **, $P < 0.01$; ns, not significant). (B) Western blot analysis of MMP3 protein expression after knockdown of P1 CMs using si-MMP3-810.

Table S1. Primer sequences used for PCR.

Gene names	Orientations	Sequences (5'-3')
IGF2BP3	F	CATCTGTTTATTCCCGCCCTGTCC
	R	TCACCATCCGCACTTTAGCATCTG
YTHDF1	F	ACAGTTACCCCTCGATGAGTG
	R	GGTAGTGAGATACGGGATGGGA
YTHDF2	F	GAGCAGAGACCAAAAGGTCAAG
	R	CTGTGGGCTCAAGTAAGGTTC
METTL3	F	CTGGGCACTTGGATTTAAGGAA
	R	TGAGAGGTGGTGTAGCAACTT
METTL14	F	CTGAGAGTGCGGATAGCATTG
	R	GAGCAGATGTATCATAGGAAGCC
WTAP	F	TAGACCCAGCGATCAACTTGT
	R	CCTGTTTGGCTATCAGGCGTA
ALKBH5	F	CGCGGTCATCAACGACTACC
	R	ATGGGCTTGAACTGGAACTTG
FTO	F	TTCATGCTGGATGACCTCAATG
	R	GCCAACTGACAGCGTTCTAAG
MMP3	F	GACGATGATGAACGATGGACAGAGG
	R	TGTGGAGGACTTGTAGACTGGGTAC
CTSS (1)	F	GCCAGCCATTCCTCCTTCTTCTTC
	R	CAAGAGTCCCATAGCCAACCACAAG
CTSS (2)	F	GCCAGCCATTCCTCCTTCTTCTTC
	R	CAAGAGTCCCATAGCCAACCACAAG
IL1RN (1)	F	CGCTTTACCTTCATCCGCTCTGAG
	R	AGGACGGTCAGCCTCTAGTGTTG
IL1RN (2)	F	CGCTTTACCTTCATCCGCTCTGAG
	R	AGGACGGTCAGCCTCTAGTGTTG
IL1RN (3)	F	CGCTTTACCTTCATCCGCTCTGAG
	R	AGGACGGTCAGCCTCTAGTGTTG
CCL6	F	ACTGCTGCTTCTCTTATGCCACAC
	R	CCCTCCTGCTGATAAAGATGATGCC
CCL9	F	GCAGTCTGAAGGCACAGCAAGG
	R	GGACAGGCAGCAATCTGAAGAGTC
MMP12 (1)	F	AGCAACTGGGCAACTGGACAAC
	R	CCGCTTCATCCATCTTGACCTCTG
MMP12 (2)	F	AGCAACTGGGCAACTGGACAAC
	R	CCGCTTCATCCATCTTGACCTCTG
MMP12 (3)	F	AGCAACTGGGCAACTGGACAAC
	R	CCGCTTCATCCATCTTGACCTCTG
LYZ2	F	CTCCTGCTTTCTGTCACTGCTCAG
	R	CCATAGTAGCCAGCCATTCCATTCC
CXCL3	F	GCCACTCTCAAGGATGGTCAAGAAG
	R	GGACTTGCCGCTCTTCAGTATCTTC

SERPINB2 (1)	F	CAAACTGGCACAAAGCTACGAACTC
	R	CGCTGGCTTGATGGAACACCTC
SERPINB2 (2)	F	CAAACTGGCACAAAGCTACGAACTC
	R	CGCTGGCTTGATGGAACACCTC
CEMIP	F	TCTGTCGCAGTGAATGATGAAGGC
	R	GCTCCAAGGGTGTCTAAATCCAAGG
SPRR1a	F	CCAGAGCCCTGCCAACCTAAGG
	R	CTTCTGCTGGTATGGTGATGGAGTG
HP (1)	F	CGGCTATGTGGAGCACTTGGTTC
	R	AGCGACTGTGTTCACCCATTGC
HP (2)	F	CTGGTGGAGATTGAGAAGGTCGTTC
	R	GAAGGCAGGCAGATAGGCATGAC
CCL3	F	CTCCCAGCCAGGTGTCATTTTCC
	R	CAGGCATTCAGTTCCAGGTCAGTG
SLCO4A1 (1)	F	GCTCATCGCAGGGCTCACTTTC
	R	AGACCATCTGAGGACACGGAAGG
SLCO4A1 (2)	F	TGAGAAGCGGAGTAGCCAGACAG
	R	GCACACAGGAAGAAGAGGAAGCC
CXCL5	F	TGCGTTGTGTTTGCTTAACCGTAAC
	R	TGACTTCCACCGTAGGGCACTG
GAPDH	F	GCCTTCCGTGTTCCTACCC
	R	CCCTCAGATGCCTGCTTCAC

Table S2. Reagents used in this study.

Reagents		Sources	Cat. numbers
Primary antibodies	Anti-METTL3	Abcam	ab195352
	Anti-FTO	CST	45980
	Anti-YTHDF1	Proteintech	17479-1-AP
	Anti-YTHDF2	Proteintech	24744-1-AP
	Anti-GAPDH	Proteintech	10494-1-AP
	Anti-IGF2BP3	Proteintech	14642-1-AP
	Anti-YTHDC1	Abcam	ab259990
	Anti-YTHDC2	Abcam	ab220160
	Anti-WTAP	CST	56501
	Anti-β-Actin	CST	4970
	Anti-Ki-67	CST	11882
	Anti-Hochest	CST	4082
	Anti-cTnT	Proteintech	15513-1-AP
	Anti-pH3	Abcam	ab80612
	CD31	Abcam	Ab76533
	TNF-α	CST	119485
	IL-1ß	CST	127035
	IL-6	CST	129125
Secondary antibodies	HRP-conjugated affinipure goat anti-mouse IgG (H+L)	Proteintech	SA00001-1
	HRP-conjugated affinipure goat anti-Rabbit IgG (H+L)	Proteintech	SA00001-2
	Alexa Fluor-488 goat anti-mouse IgG	Abcam	Ab150113
	Alexa Fluor-594 goat anti-rabbit IgG	Abcam	Ab150080
Culture medium, Kits, and other reagents	Cell-Light EdU Apollo-567 In Vitro Kit	RIBOBIO	C10310-1
	BCA Protein Assay Kit	Beyotime	P0010S
	Cell Cycle and Apoptosis Analysis Kit	Beyotime	C1052
	ROS Assay Kit	Beyotime	S0033S
	Annexin V-FITC Apoptosis Detection Kit	Beyotime	C1062S
	Actinomycin D	MCE	HY-17559
	TUNEL Apoptosis detection Kit	Beyotime	C1089
	Magna RIP™ Quad RNA-Binding Protein Immunoprecipitation Kit	Millipore	17-704
	Lipofectamine® RNAiMAX	Invitrogen	13778-150
	Fetal bovine serum	Gibco	10099-141
	DMEM	Gibco	12440079
	TTC	Sigma	T8877

Table S3. Target sequences of siRNAs and shRNAs used for gene knockdown experiments.

Names	Orientations	Oligonucleotides
Negative control	Sense	UUCUCCGAACGUGUCACGUTT
	Antisense	ACGUGACACGUUCGGAGAATT
IGF2BP3 siRNA-1	Sense	AAAUGAUAUUGCUUCCAUGAAUCUU
	Antisense	AAGAUUCAUGGAAGCAAUAUCAUUU
MMP3 siRNA-137	Sense	CCAUUGCAUGACAGUGCAATT
	Antisense	UUGCACUGUCAUGCAAUGGTT
MMP3 siRNA-810	Sense	CCACAGACUUGUCCCGUUUTT
	Antisense	AAACGGGACAAGUCUGUGGTT
MMP3 siRNA-1480	Sense	CCACAUAUUGAAGAGCAAUTT
	Antisense	AUUGCUCUUCAAUAUGUGGTT
sh-METTL3 (8746-1)	5’-3’	GCTGACCCACCTTGGGATATT
sh-METTL3 (8747-2)	5’-3’	GAAGGAACACTGCTTGGTTGG
sh-METTL3 (8748-1)	5’-3’	GCCAAGGAACAGTCCATTGTT
sh-Ctrl	5’-3’	TTCTCCGAACGTGTCACGT
